# Food deprivation among adults in India: an analysis of specific food categories, 2016–2021

**DOI:** 10.1016/j.eclinm.2023.102313

**Published:** 2023-11-20

**Authors:** Anoop Jain, Smriti Sharma, Rockli Kim, S.V. Subramanian

**Affiliations:** a Boston University School of Public Health, 715 Albany St. Boston, MA, 02118, USA; b Tata Trusts, R.K. Khanna Tennis Stadium, Africa Avenue, New Delhi, India; c Division of Health Policy & Management, College of Health Science, Korea University, 145 Anam-ro, Seongbuk-gu, Seoul 02841, South Korea; d Harvard Center for Population and Development Studies, Cambridge, MA, 02138, USA; e Department of Social and Behavioral Sciences, Harvard T.H. Chan School of Public Health, Boston, MA, 02115, USA; f Interdisciplinary Program in Precision Public Health, Department of Public Health Sciences, Graduate School of Korea University, 145 Anam-ro, Seongbuk-gu, Seoul, 02841, South Korea

**Keywords:** Food deprivation, India, Food group consumption, Adult food consumption, National Family Health Survey

## Abstract

**Background:**

Adult undernourishment remains pervasive throughout India, and often results from food deprivation, which refers to the inadequate consumption of foods with caloric and nutrient significance. Therefore, understanding the extent to which food groups are missing from an individual's diet is essential to understanding the extent to which they are undernourished.

**Methods:**

We used data from two National Family Health Surveys conducted in 2016 and 2021 for this cross-sectional analysis. The study population consisted of women and pregnant women between the ages of 15–49, and men between the ages of 15–54. We examined shifts in the percentage of people not consuming dairy, pulses/beans/legumes, dark leafy green vegetables, fruits, eggs, and fish and meat among women, pregnant women, and men between the two time points. We also examined these patterns by household wealth and education, two important markers of socioeconomic status.

**Findings:**

Overall, we found that fewer women, pregnant women, and men were not eating each of the six food groups in 2021 than in 2016. Additionally, the gap in food group consumption between women, pregnant women, and men in the lowest and highest socioeconomic groups shrank between 2016 and 2021. Yet, food group deprivation remained most prevalent among those in the lowest socioeconomic groups. The two exceptions for this were for eggs and meat/fish. Nevertheless, the majority of India's poorest and least educated adults are not consuming high-quality protein sources, including dairy, the consumption of which is far more common among wealthier and more educated Indian adults.

**Interpretation:**

Our results show that fewer adults were not consuming important food groups in 2021 than in 2016. However, many of India's poorest and least educated adults are still not consuming high-quality sources of protein or fruits, two food groups that are essential for good health. While adults might be getting protein and nutrients from pulses, legumes, beans, and other vegetables, efforts are needed to improve affordability of, and access to, high-quality sources of protein and fruits.

**Funding:**

This work was supported by the 10.13039/100000865Bill & Melinda Gates Foundation, INV- 002992.

**Research in context**

**Evidence before this study**

We searched Google Scholar to examine what, if any, prior literature exists with regards to food group consumption among adults in India. We conducted various searches, each with a different combination of key words and phrases. The first was “trends in food group consumption in India”. This yielded a number of articles with direct relevance to this study. These studies examined the major food groups being consumed by people in India, and how this relates to the prevalence of various forms of morbidity. However, many of these studies relied on older data, and did not explicitly examine changes in food group consumption between 2016 and 2021. Furthermore, these studies did not have an explicit focus on food group consumption by wealth and education. Next, we searched for “trends in dietary protein consumption India”. This search yielded a number of papers that examined the various sources of protein in the Indian diet, and how consumption of these foods has changed over time. However, none of these papers used data as current as what we used to conduct this analysis. Next, we searched for “trends in fruit and vegetable consumption India”. This search yielded studies on how fruit and vegetable consumption has changed throughout India over time. Again, these studies used outdated data.

**Added value of this study**

This paper uses the latest data from India to examine changes in the percentage of adults not consuming essential food groups in India. We also examined these changes by household wealth and education, which has not explicitly been done before. This study examines these changes from every state and Union Territory (36 total geographic units). We found that overall consumption of the six food groups included in this study increased between 2016 and 2021. We also found that the gap between those in the highest and lowest socioeconomic categories shrank during this time period. However, the majority of women, pregnant women, and men were not consuming eggs or meat and fish regardless of wealth or educational attainment.

**Implications of all the available evidence**

Sustainable Development Goal (SDG) 2.1 seeks to end hunger and ensure access to safe, nutritious and sufficient food year-round by 2030. Alleviating undernourishment, often defined by food deprivation, is the primary indicator for SDG 2.1. As other studies have shown in the past, our findings show that more Indians are eating a full complement of healthy foods than in the past. However, major dietary gaps persist, particularly for those in low socioeconomic position. Food policies in India should focus on increasing the availability and affordability of these food groups. This will help India make progress towards achieving a far more nourished population.

## Introduction

Sustainable Development Goal (SDG) 2.1 seeks to end hunger and ensure access to safe, nutritious and sufficient food year round by 2030.^1^ The primary indicator for this goal is a reduction in the prevalence of people who are experiencing undernourishment, which stems in large part from being deprived of foods that are energy and protein dense, and that have essential minerals and vitamins.^2^ For example, dairy is an important source of calcium, which is essential for bone health,^5^ and in India, is an important source of protein as well.^6^ Legumes, pulses, and beans are another important source of protein, especially for vegetarians.^5^ Fruits and vegetables are an essential source of micronutrients such as folate, vitamin A, vitamin B12. Deficiencies in these micronutrients are pervasive throughout India,^7^^,^^8^ and could help explain why anemia has worsened in some parts of India in the past five years.^9^ Similarly, meat, fish, dairy, and eggs are all important sources of protein and other essential micronutrients.^10^, ^11^, ^12^, ^13^ Being deprived of these foods undermines an individual's ability to live a healthy and productive life given their sex, age, stature, and level of physical activity.^14^ Thus, lowering the prevalence of undernourishment in order to achieve SDG 2.1 depends in large part on eliminating food and food group deprivation.

Studying undernourishment through the lens of food group deprivation in India is critically important given that India is currently not on target to meet the various dimensions of SDG 2.1. As of 2022, India ranks 107th out of 121 countries on the global hunger index,^2^ and according to the United Nations Food and Agriculture Organization, there were approximately 224 million undernourished people living throughout India as of 2021.^4^ Additionally, food insecurity, a state where a person lacks access to safe and nutritious food, either due to lack of availability or lack of resources, remains extremely prevalent throughout India.^3^

Much of the literature examining specific food group deprivation throughout India has focused on children. Recent evidence shows that many children in India are at risk of consuming no food at all.^15^ When food is available, children are more likely to consume breastmilk, grains, roots, and tubers, and/or dairy, than vitamin-A rich fruits/vegetables, legumes and nuts, meat and/or eggs, foods that are positively associated with improved child growth outcomes.^16^ Much of what children eat is associated with maternal education and other markers of socioeconomic status.^17^^,^^18^ The extent to which households are food secure is also a key determinant of what children eat.^19^ Furthermore, gender-based disparities in food group consumption extend into adolescence, as adolescent boys are more likely to consume nutritious foods than adolescent girls in some settings.^20^

Prior studies have also examined what adults are eating throughout India. However, many of these studies rely on old data. Studies conducted prior to 2010 show that the diets of Indian adults have shifted.^21^, ^22^, ^23^, ^24^ In fact, the consumption of various cereals, such as millets, has been declining while the consumption of salt, oils, and animal products has risen.^25^ And despite rising incomes, consumed energy content steadily declined throughout India between 1980 and 2010.^23^^,^^26^ Using data from 2012, Sharma et al. show that not only is the daily consumption of calories among Indian adults below what international guidelines suggest,^5^^,^^6^ the consumption of fruits, vegetables, legumes, meat, fish, and eggs is also lower than the recommended amounts.^6^ Another study examined food group consumption among adults in India using data from 2004 and 2012, showing that food group deprivation decreased during that time period.^27^

The purpose of this paper is to use recent data to understand what food groups are still not being consumed by adults throughout India. Rapid urbanization,^28^ the realities of climate change,^29^^,^^30^ and COVID-19 are some of the major forces shaping dietary patterns in India.^31^^,^^32^ As such, we use self-reported food group consumption data from India's two most recent National Family Health Surveys (NFHS) conducted in 2016 and 2021. We elucidate the percent of women, pregnant women, and men who do not consume dairy, pulses/legumes/beans, dark leafy green vegetables, vitamin-A rich fruits, eggs, and meat/fish in each survey round. Furthermore, we examine the percent of women, pregnant women, and men who do not consume these food groups by household wealth and education, two important markers of socioeconomic status. The results from this study will provide key insights into what food groups are still lacking from Indian diets, thereby illuminating the nature of hunger that persists throughout India. This can help inform policies aimed at improving nutrition outcomes.

## Methods

### Data

This analysis used data from the fourth and fifth rounds of India's National Family Health Survey (NFHS-4 and NFHS-5). NFHS-4 was conducted between 2015 and 2016, while NFHS-5 was conducted between 2019 and 2021. Both NFHS-4 and NFHS-5 used a multistage stratified cluster sampling design. The sampling frame for both surveys is based on the most recent Census of India from 2011.

### Study population

The study population included women (pregnant and non) between the ages of 15–49, and men between the ages of 15–54. NFHS-4 contains complete food consumption data from 699,686 women between the ages of 15–49, along with 32,428 pregnant women. The NFHS-4 has complete food consumption data from 112,122 men between the ages of 15–54. Additionally, this dataset contains food consumption data from 63,696 couples. Data from NFHS-5 in 2021 contains complete food consumption information from 724,115 women between the ages of 15–49, 28,408 pregnant women, and for 101,839 men between the ages of 15–54. The NFHS-5 dataset contains food consumption data from 57,693 couples.

### Outcomes

The purpose of this paper was to assess the extent of food deprivation experienced by adults in India. The EAT-Lancet commission provides guidance on which food groups need to be consumed to promote human health while accounting for regional variations in dietary preferences.^5^ We used the data made available in the NFHS to follow this guide. First, women and men were asked how frequently they eat a food group (never, daily, weekly, occasionally). We considered the ‘daily’ or ‘weekly’ responses as an indication that the food group was being consumed. However, we coded those who replied ‘occasionally’ or ‘never’ for a food group as not consuming that food item. We applied this to six food groups, which were: (a) Dairy; (b) Pulses, beans, and legumes; (c) Dark leafy green vegetables; (d) Vitamin-A rich fruits; (e) Eggs; (f) Chicken, meat, or fish. The dairy food group consisted of milk and curd. The meat and fish food group consisted broadly of chicken or other types of meat (beef, pork, or lamb), and fish (including shellfish). Each food group was dichotomized as yes or no based on the frequency of consumption.

### Statistical analysis

We estimated the weighted prevalence and 95% confidence intervals of all women (including pregnant women), pregnant women, and men not consuming each food group. The NFHS is a Demographic and Health Survey (DHS) and follows the DHS sample weighting protocol. These weights are applied to each case in order to adjust for variations in the selection probability of cases. For this analysis, we used the individual weights. We repeated this for NFHS-4 and NFHS-5. Additionally, we conducted this analysis by household wealth quintile and by educational attainment. Wealth quintiles in the NFHS are constructed by asking households about their ownership of consumer items, each of which is assigned a weight, the sum of which is used to calculate the household's wealth quintile. For education, we compare food group consumption between those with no education and those with above a 12th grade education. Data on educational attainment is obtained by directly asking respondents about how much school they completed. Using logistic regression, we estimated the odds ratios and 95% confidence intervals to assess the independent associations between wealth and education on food group consumption in each year. Additionally, we pooled the datasets to assess the association between time and food group consumption, while including an interaction term between year and wealth, and year and education. Finally, we examined gender-based differences in food group consumption among all women and men, and among wives and husbands within the same household.

### Ethics statement

NFHS data collection was approved by the International Institute for Population Studies Institutional Review Board. This analysis did not meet the regulatory definition of human subjects research as per the Harvard Longwood Campus and was thus exempt from full review.

### Role of the funding source

The funder of the study had no role in study design, data collection, data analysis, data interpretation, or writing of the report.

## Results

### Sample characteristics

Our sample contained food consumption data from all 640 districts and 36 states/union territories in India from 2016. We included food consumption data from all 707 districts and 36 states/union territories in India from 2021. In 2016, the median age of women was 29, and 24 among pregnant women. In 2016, the median age of men was 30. In 2021, the median age of women was 30, and 25 among pregnant women. In 2021, the median age of men was 31. In terms of education, however, we found that the share of women, regardless of pregnancy status, with no education decreased, while the share of women with an education above the 12th grade increased between 2016 and 2021. These results are presented in Table 1 below.

**Table 1:** Age, wealth, and education characteristics of study populations in 2016 and 2021.

	2016			2021		
	All women	Pregnant women	All men	All women	Pregnant women	All men
**Age groups**						
15–19	124,878 (17.9%)	3686 (11.4%)	19,082 (17.0%)	122,480 (16.9%)	2985 (10.5%)	16,657 (16.4%)
20–24	122,955 (17.6%)	13,735 (42.4%)	16,630 (14.8%)	118,700 (16.4%)	11,184 (39.4%)	14,413 (14.2%)
25–29	115,076 (16.5%)	9813 (30.3%)	16,151 (14.4%)	118,379 (16.4%)	9167 (32.3%)	14,360 (14.1%)
30–34	97,048 (13.9%)	3654 (11.3%)	14,640 (13.1%)	101,049 (13.9%)	3601 (12.7%)	13,292 (13.1%)
35–39	90,433 (12.9%)	1215 (3.8%)	13,897 (12.4%)	98,068 (13.5%)	1216 (4.3%)	12,874 (12.6%)
40–44	76,627 (10.9%)	254 (0.8%)	11,954 (10.7%)	81,380 (11.2%)	208 (0.7%)	10,838 (10.6%)
45–49	72,669 (10.4%)	71 (0.2%)	11,171 (9.9%)	84,059 (11.6%)	47 (0.2%)	10,833 (10.6%)
50–54	–	–	8597 (7.7%)	–	–	8572 (8.4%)
**Wealth quintiles**						
Lowest	133,249 (19.0%)	7683 (23.7%)	18,412 (16.4%)	149,844 (20.7%)	6932 (24.4%)	19,796 (19.4%)
Low	149,466 (21.4%)	7616 (23.5%)	23,220 (20.7%)	160,340 (22.1%)	6591 (23.2%)	22,599 (22.2%)
Middle	147,168 (21.0%)	6597 (20.3%)	24,331 (21.7%)	151,505 (20.9%)	5745 (20.2%)	21,715 (21.3%)
High	138,502 (19.8%)	5575 (17.2%)	23,383 (20.9%)	139,607 (19.3%)	5086 (17.9%)	20,209 (19.8%)
Highest	131,301 (18.8%)	4957 (15.3%)	22,776 (20.3%)	122,819 (16.9%)	4054 (14.3%)	17,520 (17.2%)
**Educational attainment**						
No education	196,556 (28.1%)	8149 (25.1%)	15,007 (13.4%)	167,304 (23.1%)	4653 (16.4%)	12,269 (12.1%)
Primary (1st–5th grade)	88,290 (12.6%)	4280 (13.2%)	14,351 (12.8%)	84,983 (11.7%)	3087 (10.9%)	11,710 (11.5%)
Secondary (6th–12th grade)	334,927 (47.9%)	16,074 (49.6%)	65,260 (58.2%)	370,012 (51.1%)	10,005 (56.3%)	60,018 (58.9%)
Higher (above 12th grade)	79,913 (11.4%)	3925 (12.1%)	17,504 (15.6%)	101,816 (14.1%)	4663 (16.4%)	17,842 (17.5%)

### Overall patterns in food groups not being consumed

**Fig. 1:**
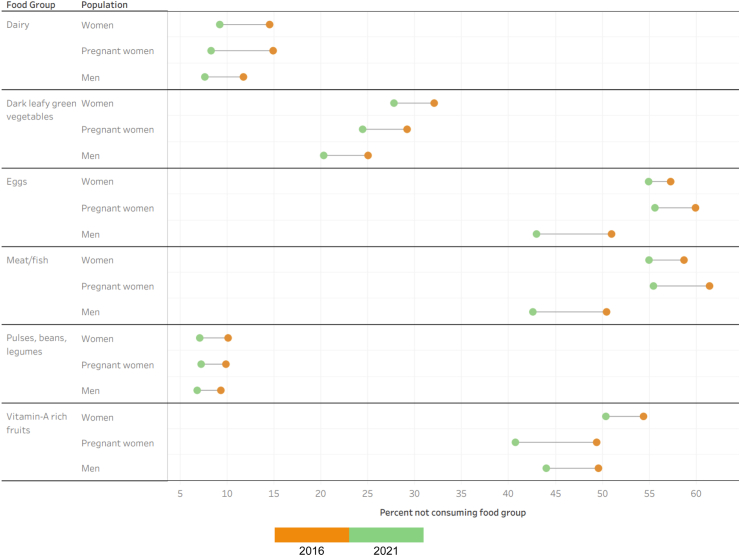
Overall changes in food group deprivation between 2016 and 2021 among women, pregnant women, and men.

Overall, we found that the percentage of women, pregnant women, and men not consuming dairy declined between 2016 and 2021. This was also true for pulses/legumes/beans. For this food group, the percentage of women, pregnant women, and men not consuming was below 10% by 2021. We found the same patterns for dark leafy green vegetable consumption for women, pregnant women, and men. The percentage of women, pregnant women, and men not consuming vitamin-A rich fruits also decreased between 2016 and 2021. However, just over 50% of women were still not eating this food group as of 2021, while the percent of pregnant women and men not consuming the food group was 40.7% and 44%, respectively, as of 2021. The percent of women, pregnant women, and men not consuming eggs decreased between 2016 and 2021. However, over 50% of women and pregnant women were still not consuming this food group as of 2021, while the percent of men not consuming eggs was 42.6% in 2021. We found the same patter to be true for meat/fish consumption. These results are presented in Table 2 and Fig. 1.

**Table 2:** Percent not consuming food group with 95% confidence intervals for women, pregnant women, and men in 2016 and 2021.

	All women		Pregnant women		Men	
	2016	2021	2016	2021	2016	2021
Dairy	32 (31.9–32.2)	27.8 (27.7–27.9)	29.2 (28.7–29.7)	24.4 (23.9–24.9)	25 (24.7–25.2)	20.3 (20–20.5)
Pulses/legumes/beans	10.1 (10–10.2)	7.1 (7–7.1)	9.9 (9.5–10.2)	7.2 (6.9–7.5)	9.3 (9.1–9.5)	6.8 (6.6–7)
Dark leafy green vegetables	14.5 (14.4–14.6)	9.2 (9.1–9.3)	14.9 (14.5–15.3)	8.3 (8–8.6)	11.7 (11.5–11.9)	7.6 (7.5–7.8)
Vitamin-A rich fruits	54.3 (54.2–54.5)	50.3 (50.2–50.4)	49.3 (48.8–49.9)	40.7 (40.1–41.3)	49.5 (49.2–49.8)	44 (43.7–44.3)
Eggs	58.6 (58.5–58.8)	54.9 (54.8–55)	61.4 (60.8–61.9)	55.4 (54.8–56)	50.4 (50.1–50.7)	42.6 (42.2–42.9)
Meat/fish	57.2 (57.1–57.3)	54.9 (54.8–55)	59.9 (59.3–60.4)	55.6 (55–56.1)	50.9 (50.6–51.2)	42.9 (42.6–43.2)

These values were used to construct Fig. 1.

### Patterns in food groups not being consumed by wealth and education

**Fig. 2:**
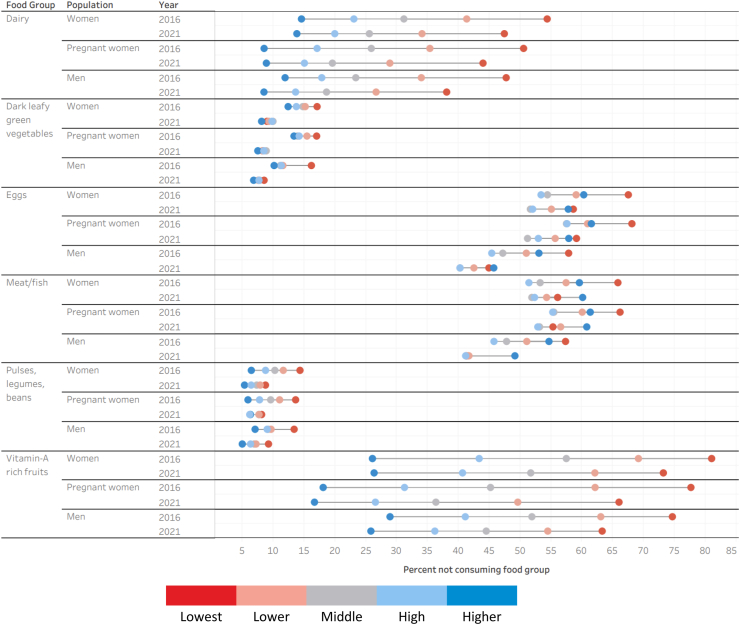
Percentage of women, pregnant women, and men not consuming a food group by wealth quintile.

Overall, we found that the gap in not consuming between women, pregnant women, and men in the lowest and highest wealth quintile groups decreased for every food group between 2016 and 2021. However, we found that more women, pregnant women, and men were not consuming each food group than their counterparts in higher wealth households. There were two exceptions to this. First, as of 2021, more men in the lowest wealth quintile were eating eggs than men in the highest wealth quintile. Second, by 2021, more women, pregnant women, and men in the highest wealth quintile were not eating meat/fish than women, pregnant women, and men in the lowest wealth quintile. These results are presented in Fig. 2 and Supplementary Tables S1 and S2.

**Fig. 3:**
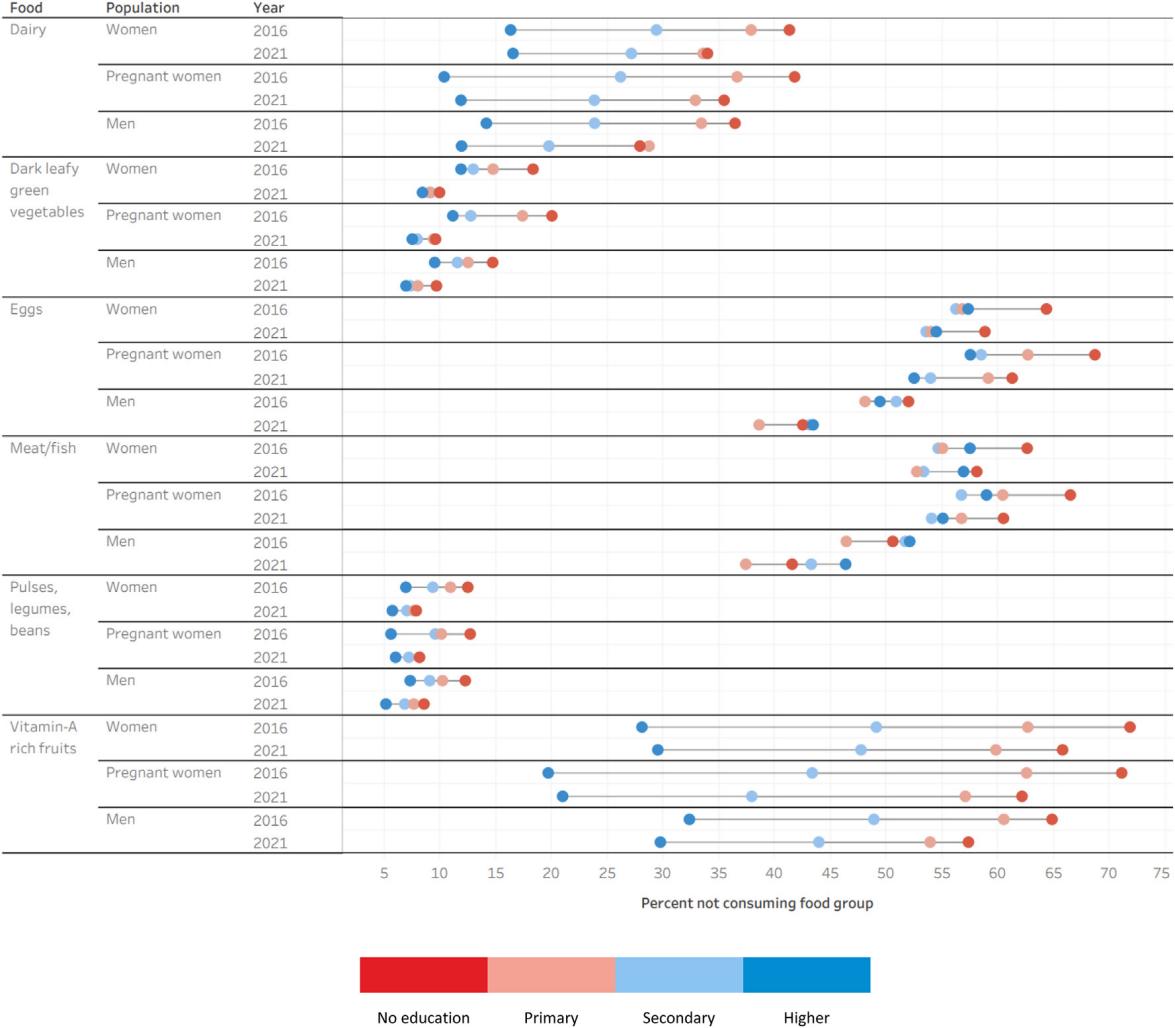
Percentage of women, pregnant women, and men not consuming a food group by educational attainment.

We found that the gap in not consuming between women, pregnant women, and men with no education and higher education fell for every food group between 2016 and 2021. As was the case when examining these patterns by wealth, fewer people with higher education were not consuming each food group than those with no education. Again, there were two exceptions to this. More men with a higher education were not eating eggs than men in the lowest education category as of 2021. Similarly, more men with a higher education were not eating meat or fish than men with just a primary education in 2016. This gap widened by over three percentage points by 2021. These results are presented in Fig. 3 and Supplementary Tables S3 and S4.

### Regression based inferences

Overall, we found that when compared to those in the highest wealth quintile, those in the lowest, low, middle, and high wealth quintiles all had statistically significant higher odds of not eating dairy, pulses/legumes/beans, dark leafy green vegetables, and vitamin-A rich fruits. Those with no education, primary, or secondary education had statistically significant higher odds of not eating those same food groups when compared to those with higher education. However, those in the lowest, low, middle, and high wealth quintiles all had lower odds of not eating eggs and meat/fish than those in the highest wealth quintile. We did not find a clear pattern of association between education and these food groups. These wealth and education associations were true for women, pregnant women, and men in both 2016 and 2021, and can be seen in Supplementary Tables S5–S7. After pooling the datasets, we found that the odds of not eating all six food groups was higher in 2016 than in 2021 for women, pregnant women, and men. The only exception to this was for meat/fish consumption among men. The odds of not eating meat/fish were slightly lower in 2016 than in 2021 for men. Furthermore, we found statistically significant associations when interacting survey year with wealth/education categories for some of the food groups. This indicates that the change in food group consumption among those in lower socioeconomic groups was different than the change experienced by those in the highest wealth and education groups. Yet, these effects, even when statistically significant, were quite small. These results are presented in Supplementary Tables S8–S10.

### Patterns in food groups not being consumed by gender

Overall, we found that the percentage of men not consuming a food group was lower than the percentage of women not consuming a food group. This was true for all six food groups in both 2016 and 2021. The widest gaps were for dairy, eggs, and meat/fish. We examined the extent to which these differences were also true among married couples. Overall, we found that the percentage of husbands not consuming a food group was lower than the percentage of wives not consuming a food group. This was true for all six food groups in both 2016 and 2021. However, we did find that the gender gap was smaller among married couples than for women and men in the overall population for dairy, eggs, and meat/fish consumption in both 2016 and 2021. We show that these patterns are largely the same when examined by household wealth and education. There are two exceptions to this. First, among those with no formal education, more men and husbands were not eating pulses/legumes/beans than women and wives in 2021. Second, the gap in egg and meat/fish consumption between women and men in the lowest socioeconomic categories is larger than the gap between wives and their husbands in the same socioeconomic groups in both 2016 and 2021. These results are presented in Table 3 and Supplementary Tables S11 and S12.

**Table 3:** Percent (95% confidence interval) of all women and men and husbands/wives not consuming a food group.

	Women	Men	Difference (p-value)	Wives	Husbands	Difference (p-value)
**2016**						
Dairy	32 (31.9–32.2)	25 (24.7–25.2)	7.0 (p < 0.01)	31.1 (30.8–31.5)	25.8 (25.4–26.1)	5.3 (p < 0.01)
Pulses/legumes/beans	10.1 (10–10.2)	9.3 (9.1–9.5)	0.8 (p < 0.01)	9.8 (9.6–10)	8.9 (8.6–9.1)	0.9 (p < 0.01)
Dark leafy green vegetables	14.5 (14.4–14.6)	11.7 (11.5–11.9)	2.8 (p < 0.01)	14.2 (13.9–14.4)	11.2 (11–11.4)	3.0 (p < 0.01)
Vitamin-A rich fruits	54.3 (54.2–54.5)	49.5 (49.2–49.8)	4.8 (p < 0.01)	53.8 (53.4–54.2)	51 (50.6–51.4)	2.8 (p < 0.01)
Eggs	58.6 (58.5–58.8)	50.4 (50.1–50.7)	8.2 (p < 0.01)	58.4 (58.1–58.8)	50.5 (50.1–50.9)	7.9 (p < 0.01)
Meat/fish	57.2 (57.1–57.3)	50.9 (50.6–51.2)	6.3 (p < 0.01)	56.4 (56–56.8)	50.5 (50.1–50.9)	5.9 (p < 0.01)
**2021**						
Dairy	27.8 (27.7–27.9)	20.3 (20–20.5)	7.5 (p < 0.01)	28.1 (27.7–28.4)	22 (21.6–22.3)	6.1 (p < 0.01)
Pulses/legumes/beans	7.1 (7–7.1)	6.8 (6.6–7)	0.3 (p < 0.01)	6.8 (6.6–7)	6.3 (6.1–6.5)	0.5 (p = 0.07)
Dark leafy green vegetables	9.2 (9.1–9.3)	7.6 (7.5–7.8)	1.6 (p < 0.01)	8.7 (8.5–8.9)	7.3 (7.1–7.5)	1.4 (p < 0.01)
Vitamin-A rich fruits	50.3 (50.2–50.4)	44 (43.7–44.3)	6.3 (p < 0.01)	50.5 (50.1–50.9)	46.9 (46.5–47.3)	3.6 (p < 0.01)
Eggs	54.9 (54.8–55)	42.6 (42.2–42.9)	12.3 (p < 0.01)	54.3 (53.9–54.8)	47.2 (46.8–47.6)	7.1 (p < 0.01)
Meat/fish	54.9 (54.8–55)	42.9 (42.6–43.2)	12.0 (p < 0.01)	53.5 (53.1–53.9)	49.2 (48.7–49.6)	4.3 (p < 0.01)

## Discussion

Our study had three salient findings. First, we found that the percentage of women, pregnant women, and men not consuming each of the six food groups fell between 2016 and 2021. However, as of 2021, the majority of women and pregnant women were not eating eggs or meat and fish. Second, the gap in food group consumption between women, pregnant women, and men in the lowest and highest socioeconomic groups shrunk between 2016 and 2021. Yet as of 2021, more women, pregnant women, and men in the lowest wealth quintile were eating meat and fish than their counterparts in the highest wealth quintile. The same was true for men when we examined the percentage of not consuming food groups by education. Third, we found that overall, fewer men and husbands were not consuming each food group than women and wives, an indication of gender-based inequity in food consumption.

There are several data limitations associated with this study. First, mothers were asked how frequently they eat their food groups (never, daily, weekly, occasionally). For the purposes of this study, we considered a food group to be consumed if the respondent said the eat it either daily or weekly. Thus, our estimates do not include consumption if it occurred less frequently than weekly. This prohibits us from properly accounting for seasonal variations in dietary patterns, an issue in many studies that examine dietary intake. Further, the dietary guidelines are recommended for daily consumption practices and weekly assessment of food consumption does not give a complete picture daily food deprivations. Future research should examine diets over different periods of time to understand variations by day of the week and/or seasons. Second, food consumption data were collected by asking respondents to recall what foods they ate. As such, the results could be potentially impacted by recall bias. Third, the NFHS does not contain any data regarding the quantities of food being consumed. Fourth, the NFHS does not capture the full set of ten food groups recommended in the construction of dietary diversity scores. The NFHS also does not collect data on many local food items being consumed that might be high in protein or nutrients.

Our results are policy relevant for several reasons. Following a recommended healthy diet can cost at least USD 5 per person per day in India.^33^ Dairy, eggs, fish, and meat—all of which are important and high-quality sources of protein—account for a large portion of this cost.^33^ The fact that these items are expensive could explain our findings showing that over 40% of India's poorest women and pregnant women are not consuming dairy, and that almost 60% of them are not consuming eggs, meat, or fish. The percentage of poor men not consuming these food groups was also quite high, around 40%. Even within households, more wives are not consuming dairy, eggs, and meat/fish than their husbands, an indication of how scarce resources are allocated. Furthermore, egg and meat consumption is a cultural flashpoint as some municipal governments have banned the display of meat, fish, and eggs on the street,^34^ and is generally more common among poorest and lower caste households, as shown in this analysis and prior studies.^35^ That egg and meat consumption is a cultural taboo could help explain why those in the highest socioeconomic categories of our study were more likely to not consume these food groups than their poorer and less educated counterparts. Nevertheless, adults, particularly as they age, need protein in order to prevent the loss of muscle and to manage their weight.^36^, ^37^, ^38^ Protein consumption during pregnancy is also extremely important for the mother and child's health, and is associated with a decreased risk of low birth weight and small-for-gestational-age births.^39^^,^^40^ While our results show that adults, regardless of household wealth, are likely consuming some protein from pulses, legumes, and beans, food subsidies are required to make other sources of protein more affordable and accessible.^41^ High dairy consumption among wealthier and more educated Indians, for example, has been shown to make up for the possible negative health effects of not consuming non-vegetarian forms of protein.^35^ Future research should also examine the viability of plant-based protein foods in the Indian context as means of addressing protein deprivation given the cultural and religious preferences for vegetarianism. Plant-based protein is also more financially and environmentally sustainable, both of which are important factors to consider.^42^^,^^43^

Prior studies have also shown the extent to which diets vary by region within India, with fruit and vegetable consumption being more common in the north and west.^25^^,^^44^ When examined through the lens of socioeconomic status, however, we show that as of 2021, the percentage of those not consuming vegetables in the lowest wealth and education categories is similar to the percentage of those not consuming vegetables in the highest wealth and education categories. In fact, our results show that the percentage of India's poorest and least educated not consuming vegetables fell between 2016 and 2021, an encouraging sign in terms of nutrition and health. However, the majority of India's poorest and least educated adults are not consuming vitamin-A rich fruits. This is important given that almost 66% of people in India are experiencing some form of micronutrient deficiency, stemming from inadequate consumption of foods rich in iron and vitamin A.^45^ Nutrition education is certainly a piece of the puzzle when trying to promote the dietary intake of nutrient rich fruits. However, strengthening food systems will also play a pivotal role in this regard. For example, investments in cold storage facilities that minimize post-harvest losses of perishable items, such as fruits, is key throughout India.^33^ India's government also needs to improve its process of procurement for these particular types of food items, while ensuring fair pay to farmers and producers.^33^

In conclusion, our results show that fewer adults were not consuming important food groups in 2021 than in 2016. However, many of India's poorest and least educated adults are still not consuming high-quality sources of protein or fruits, two food groups that are essential for good health. While adults might be getting protein and nutrients from pulses, legumes, beans, and other vegetables, efforts are needed to improve affordability of, and access to, high-quality sources of protein and fruits.

## Contributors

Conceptualization and Design: AJ, RK, SVS.

Data Acquisition and Analysis: AJ.

Data Interpretation: AJ, SS, RK, SVS.

Drafting of the Manuscript: AJ.

Critical revisions to Manuscript: AJ, SS, RK, SVS.

Overall Supervision: RK, SVS.

## Data sharing statement

The datasets generated during and/or analyzed during the current study are available from the corresponding author on reasonable request.

## Declaration of interests

We declare no competing interests.
