# Corrigendum to—“Food deprivation among adults in India: an analysis of specific food-categories, 2016–2021” [eClinical medicine 66(2023) 102313]

**DOI:** 10.1016/j.eclinm.2024.102488

**Published:** 2024-02-14

**Authors:** Anoop Jain, Smriti Sharma, Rockli Kim, S.V. Subramanian

**Affiliations:** a Boston University School of Public Health, 715 Albany St, Boston, MA 02118, USA; b Tata Trusts, R.K. Khanna Tennis Stadium, Africa Avenue, New Delhi, India; c Interdisciplinary Program in Precision Public Health, Department of Public Health Sciences, Graduate School of Korea University, 145 Anam-ro, Seongbuk-gu, Seoul, 02841, South Korea; d Division of Health Policy & Management, College of Health Science, Korea University, 145 Anam-ro, Seongbuk-gu, Seoul 02841, South Korea; e Harvard Center for Population and Development Studies, Cambridge, MA 02138, USA; f Department of Social and Behavioral Sciences, Harvard T.H. Chan School of Public Health, Boston, MA 02115, USA

We regret that the published article titled, “Food deprivation among adults in India: an analysis of specific food group categories, 2016–2021” contained a minor error in Figure 1. This error does not alter the findings or conclusion published in our original paper.

More specifically, the values in Figure 1 should correspond with the values in Table 2. However, there was a mistake made when creating the figure such that the food group categories and values were accidently switched for Dairy and Dark Leafy Green Vegetables and Eggs and Meat/Fish. Again, this error does not alter any of the findings or conclusions as the values in Table 2 are correct. The correct version of the figure is below and can been seen in “Corrected Fig. 1”. As is evident, this change makes no consequential difference whatsoever to the results and interpretation drawn from the original study.

**Corrected Fig. 1:**
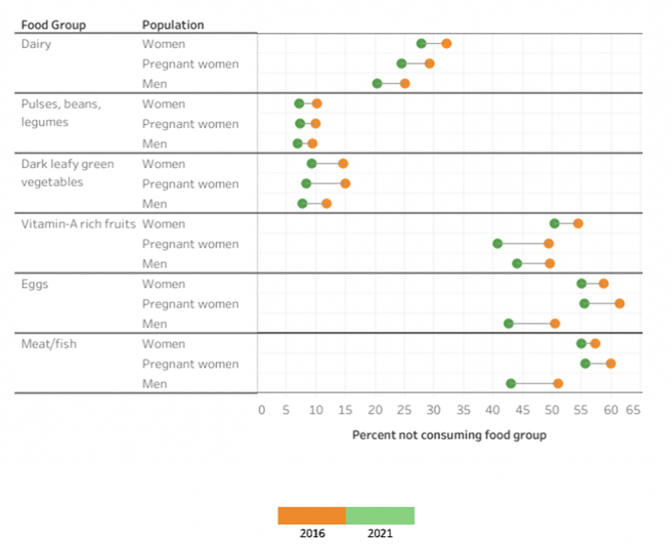
Overall changes in food group deprivation between 2016 and 2021 among women, pregnant women, and men.

