# Supplementary material for: Food deprivation among adults in India: an analysis of specific food categories, 2016–2021
Source: eClinicalMedicine. 2023 Nov 20;66:102313. doi: 10.1016/j.eclinm.2023.102313 (PMC10679480; doi:10.1016/j.eclinm.2023.102313)
Supplement: Supplementary Tables [file mmc1.docx]

**Supplementary table 1:** Percent not consuming (95% confidence intervals) each food group by wealth quintile in 2016

|  | **Wealth quintile** | **Women** | **Pregnant women** | **Men** |
| --- | --- | --- | --- | --- |
| **Dairy** | **Lowest** | 54.36 (54.09-54.63) | 50.53 (49.42-51.65) | 47.71 (46.99-48.44) |
|  | **Low** | 41.32 (41.07-41.57) | 35.36 (34.28-36.43) | 33.97 (33.36-34.58) |
|  | **Middle** | 31.14 (30.9-31.37) | 25.85 (24.79-26.91) | 23.34 (22.8-23.87) |
|  | **High** | 23.03 (22.8-23.25) | 17.06 (16.08-18.05) | 17.82 (17.33-18.31) |
|  | **Highest** | 14.53 (14.34-14.72) | 8.48 (7.71-9.26) | 11.88 (11.46-12.3) |
| **Pulses/legumes/beans** | **Lowest** | 14.29 (14.11-14.48) | 13.59 (12.82-14.35) | 13.34 (12.85-13.83) |
|  | **Low** | 11.58 (11.42-11.74) | 10.99 (10.29-11.69) | 9.61 (9.23-9.99) |
|  | **Middle** | 10.22 (10.06-10.37) | 9.55 (8.84-10.25) | 9.17 (8.8-9.53) |
|  | **High** | 8.72 (8.57-8.87) | 7.74 (7.04-8.45) | 8.98 (8.61-9.34) |
|  | **Highest** | 6.41 (6.28-6.55) | 5.87 (5.21-6.52) | 7.02 (6.69-7.36) |
| **Dark leafy green vegetables** | **Lowest** | 17.06 (16.86-17.27) | 16.99 (16.15-17.83) | 16.14 (15.61-16.67) |
|  | **Low** | 15.15 (14.96-15.33) | 15.42 (14.61-16.23) | 11.53 (11.12-11.94) |
|  | **Middle** | 14.75 (14.57-14.93) | 13.86 (13.03-14.7) | 11.11 (10.71-11.5) |
|  | **High** | 13.7 (13.52-13.88) | 14.16 (13.24-15.07) | 11.26 (10.85-11.67) |
|  | **Highest** | 12.37 (12.19-12.55) | 13.32 (12.37-14.27) | 10.1 (9.71-10.49) |
| **Vitamin-A rich fruits** | **Lowest** | 81.02 (80.8-81.23) | 77.66 (76.73-78.59) | 74.64 (74.01-75.27) |
|  | **Low** | 69.14 (68.91-69.37) | 62.12 (61.03-63.21) | 63.05 (62.43-63.67) |
|  | **Middle** | 57.46 (57.21-57.71) | 45.18 (43.98-46.38) | 51.88 (51.25-52.51) |
|  | **High** | 43.34 (43.08-43.6) | 31.24 (30.02-32.45) | 41.08 (40.45-41.71) |
|  | **Highest** | 26.03 (25.8-26.27) | 18.05 (16.98-19.12) | 28.88 (28.3-29.47) |
| **Eggs** | **Lowest** | 67.51 (67.25-67.76) | 68.09 (67.04-69.13) | 57.84 (57.12-58.55) |
|  | **Low** | 59.05 (58.8-59.3) | 60.9 (59.81-62) | 50.98 (50.33-51.62) |
|  | **Middle** | 54.4 (54.14-54.65) | 57.49 (56.29-58.68) | 47.16 (46.53-47.79) |
|  | **High** | 53.36 (53.1-53.63) | 57.57 (56.27-58.87) | 45.37 (44.74-46.01) |
|  | **Highest** | 60.28 (60.02-60.55) | 61.52 (60.17-62.88) | 53.02 (52.37-53.67) |
| **Meat/fish** | **Lowest** | 65.82 (65.57-66.08) | 66.17 (65.11-67.23) | 57.33 (56.62-58.05) |
|  | **Low** | 57.42 (57.17-57.67) | 60.04 (58.94-61.14) | 51.05 (50.41-51.7) |
|  | **Middle** | 53.22 (52.96-53.47) | 55.45 (54.25-56.65) | 47.78 (47.15-48.41) |
|  | **High** | 51.4 (51.13-51.66) | 55.26 (53.95-56.56) | 45.73 (45.09-46.37) |
|  | **Highest** | 59.58 (59.32-59.85) | 61.34 (59.98-62.7) | 54.65 (54-55.29) |

**Supplementary table 2:** Percent not consuming (95% confidence intervals) each food group by wealth quintile in 2021

|  | **Wealth quintile** | **Women** | **Pregnant women** | **Men** |
| --- | --- | --- | --- | --- |
| **Dairy** | **Lowest** | 47.42 (47.16-47.67) | 43.96 (42.79-45.13) | 38.07 (37.4-38.75) |
|  | **Low** | 34.08 (33.85-34.31) | 28.86 (27.77-29.95) | 26.63 (26.05-27.2) |
|  | **Middle** | 25.54 (25.32-25.76) | 19.55 (18.52-20.57) | 18.59 (18.08-19.11) |
|  | **High** | 19.93 (19.72-20.14) | 15.01 (14.03-15.99) | 13.58 (13.1-14.05) |
|  | **Highest** | 13.79 (13.59-13.98) | 8.85 (7.98-9.72) | 8.46 (8.05-8.87) |
| **Pulses/legumes/beans** | **Lowest** | 8.71 (8.56-8.85) | 8.08 (7.44-8.72) | 9.19 (8.79-9.6) |
|  | **Low** | 7.85 (7.72-7.98) | 7.67 (7.03-8.31) | 7.18 (6.84-7.51) |
|  | **Middle** | 7.28 (7.15-7.42) | 7.64 (6.96-8.33) | 6.87 (6.53-7.2) |
|  | **High** | 6.39 (6.26-6.52) | 6.15 (5.49-6.81) | 6.29 (5.95-6.62) |
|  | **Highest** | 5.31 (5.19-5.44) | 6.27 (5.52-7.01) | 4.95 (4.63-5.27) |
| **Dark leafy green vegetables** | **Lowest** | 8.95 (8.8-9.09) | 8.25 (7.6-8.89) | 8.48 (8.1-8.87) |
|  | **Low** | 9.25 (9.11-9.4) | 8.28 (7.62-8.95) | 7.51 (7.16-7.85) |
|  | **Middle** | 9.91 (9.76-10.07) | 8.83 (8.1-9.56) | 7.78 (7.43-8.14) |
|  | **High** | 9.76 (9.6-9.92) | 8.44 (7.68-9.21) | 7.65 (7.29-8.02) |
|  | **Highest** | 8.08 (7.93-8.23) | 7.47 (6.66-8.28) | 6.79 (6.41-7.16) |
| **Vitamin-A rich fruits** | **Lowest** | 73.18 (72.96-73.4) | 66 (64.89-67.12) | 63.27 (62.6-63.95) |
|  | **Low** | 62.09 (61.85-62.33) | 49.59 (48.38-50.8) | 54.45 (53.8-55.1) |
|  | **Middle** | 51.69 (51.44-51.95) | 36.33 (35.08-37.57) | 44.48 (43.82-45.14) |
|  | **High** | 40.64 (40.38-40.9) | 26.51 (25.3-27.73) | 36.16 (35.5-36.82) |
|  | **Highest** | 26.3 (26.06-26.55) | 16.64 (15.49-17.79) | 25.79 (25.14-26.43) |
| **Eggs** | **Lowest** | 58.59 (58.34-58.84) | 59.11 (57.95-60.26) | 44.91 (44.22-45.6) |
|  | **Low** | 55.04 (54.8-55.29) | 55.65 (54.45-56.85) | 42.48 (41.84-43.13) |
|  | **Middle** | 51.63 (51.38-51.88) | 51.17 (49.88-52.47) | 40.23 (39.58-40.88) |
|  | **High** | 52.01 (51.74-52.27) | 52.92 (51.55-54.3) | 40.24 (39.56-40.91) |
|  | **Highest** | 57.77 (57.5-58.05) | 57.86 (56.34-59.38) | 45.7 (44.96-46.44) |
| **Meat/fish** | **Lowest** | 56.05 (55.8-56.3) | 55.29 (54.12-56.46) | 41.22 (40.54-41.91) |
|  | **Low** | 54.27 (54.02-54.51) | 56.54 (55.35-57.74) | 41.68 (41.03-42.32) |
|  | **Middle** | 51.87 (51.62-52.12) | 53.12 (51.83-54.41) | 41.51 (40.85-42.16) |
|  | **High** | 52.32 (52.06-52.58) | 52.84 (51.46-54.21) | 41.14 (40.46-41.82) |
|  | **Highest** | 60.11 (59.83-60.38) | 60.77 (59.27-62.28) | 49.13 (48.39-49.87) |

**Supplementary table 3:** Percent not consuming (95% confidence intervals) each food group by education in 2016

|  | **Education category** | **Women** | **Pregnant women** | **Men** |
| --- | --- | --- | --- | --- |
| **Dairy** | **No education** | 41.3 (41.08-41.51) | 41.76 (40.69-42.83) | 36.42 (35.65-37.19) |
|  | **Primary** | 37.86 (37.54-38.18) | 36.61 (35.17-38.05) | 33.41 (32.64-34.18) |
|  | **Secondary** | 29.38 (29.23-29.54) | 26.16 (25.48-26.84) | 23.84 (23.51-24.16) |
|  | **Higher** | 16.31 (16.05-16.57) | 10.35 (9.4-11.31) | 14.14 (13.62-14.65) |
| **Pulses/legumes/beans** | **No education** | 12.47 (12.33-12.62) | 12.69 (11.97-13.41) | 12.25 (11.73-12.78) |
|  | **Primary** | 10.92 (10.72-11.13) | 10.11 (9.2-11.01) | 10.21 (9.71-10.71) |
|  | **Secondary** | 9.34 (9.24-9.44) | 9.56 (9.1-10.01) | 9.06 (8.84-9.28) |
|  | **Higher** | 6.92 (6.75-7.1) | 5.59 (4.87-6.31) | 7.31 (6.93-7.7) |
| **Dark leafy green vegetables** | **No education** | 18.32 (18.15-18.49) | 20.01 (19.14-20.88) | 14.7 (14.14-15.27) |
|  | **Primary** | 14.74 (14.5-14.97) | 17.37 (16.24-18.51) | 12.49 (11.95-13.04) |
|  | **Secondary** | 12.96 (12.85-13.08) | 12.74 (12.23-13.26) | 11.55 (11.3-11.79) |
|  | **Higher** | 11.87 (11.64-12.09) | 11.13 (10.15-12.12) | 9.51 (9.08-9.94) |
| **Vitamin-A rich fruits** | **No education** | 71.82 (71.62-72.02) | 71.07 (70.09-72.06) | 64.82 (64.06-65.59) |
|  | **Primary** | 62.64 (62.33-62.96) | 62.54 (61.09-63.99) | 60.5 (59.7-61.3) |
|  | **Secondary** | 49.08 (48.91-49.25) | 43.34 (42.57-44.1) | 48.86 (48.48-49.25) |
|  | **Higher** | 28.08 (27.77-28.39) | 19.68 (18.43-20.92) | 32.34 (31.65-33.03) |
| **Eggs** | **No education** | 64.32 (64.11-64.53) | 68.67 (67.67-69.68) | 51.97 (51.17-52.77) |
|  | **Primary** | 56.79 (56.46-57.11) | 62.66 (61.21-64.1) | 48.07 (47.26-48.89) |
|  | **Secondary** | 56.21 (56.04-56.38) | 58.47 (57.71-59.24) | 50.86 (50.48-51.25) |
|  | **Higher** | 57.31 (56.97-57.65) | 57.51 (55.96-59.05) | 49.41 (48.66-50.15) |
| **Meat/fish** | **No education** | 62.59 (62.38-62.81) | 66.47 (65.45-67.5) | 50.56 (49.76-51.36) |
|  | **Primary** | 55.01 (54.69-55.34) | 60.41 (58.94-61.87) | 46.39 (45.57-47.21) |
|  | **Secondary** | 54.63 (54.46-54.79) | 56.71 (55.94-57.47) | 51.67 (51.29-52.06) |
|  | **Higher** | 57.47 (57.13-57.81) | 58.95 (57.41-60.49) | 52.07 (51.33-52.81) |

**Supplementary table 4:** Percent not consuming (95% confidence intervals) each food group by education in 2021

|  | **Education category** | **Women** | **Pregnant women** | **Men** |
| --- | --- | --- | --- | --- |
| **Dairy** | **No education** | 33.96 (33.73-34.18) | 35.45 (34.07-36.82) | 27.89 (27.1-28.69) |
|  | **Primary** | 33.56 (33.24-33.88) | 32.87 (31.21-34.53) | 28.72 (27.9-29.54) |
|  | **Secondary** | 27.13 (26.99-27.28) | 23.81 (23.15-24.47) | 19.74 (19.42-20.06) |
|  | **Higher** | 16.52 (16.3-16.75) | 11.87 (10.94-12.79) | 11.92 (11.44-12.39) |
| **Pulses/legumes/beans** | **No education** | 7.86 (7.73-7.99) | 8.15 (7.36-8.94) | 8.55 (8.06-9.05) |
|  | **Primary** | 7.66 (7.48-7.83) | 8.13 (7.17-9.1) | 7.64 (7.16-8.12) |
|  | **Secondary** | 7.01 (6.93-7.1) | 7.19 (6.79-7.59) | 6.83 (6.62-7.03) |
|  | **Higher** | 5.72 (5.57-5.86) | 6 (5.32-6.69) | 5.14 (4.81-5.46) |
| **Dark leafy green vegetables** | **No education** | 9.94 (9.8-10.08) | 9.59 (8.74-10.43) | 9.67 (9.14-10.19) |
|  | **Primary** | 9.05 (8.86-9.24) | 9.41 (8.38-10.44) | 7.99 (7.5-8.48) |
|  | **Secondary** | 9.15 (9.06-9.24) | 7.94 (7.52-8.36) | 7.34 (7.14-7.55) |
|  | **Higher** | 8.41 (8.24-8.59) | 7.5 (6.74-8.26) | 6.93 (6.56-7.31) |
| **Vitamin-A rich fruits** | **No education** | 65.76 (65.54-65.99) | 62.13 (60.74-63.53) | 57.33 (56.46-58.21) |
|  | **Primary** | 59.79 (59.46-60.12) | 57.05 (55.3-58.8) | 53.9 (53-54.8) |
|  | **Secondary** | 47.71 (47.55-47.87) | 37.93 (37.17-38.68) | 43.93 (43.54-44.33) |
|  | **Higher** | 29.51 (29.23-29.79) | 20.97 (19.8-22.14) | 29.74 (29.07-30.41) |
| **Eggs** | **No education** | 58.81 (58.58-59.05) | 61.25 (59.85-62.65) | 42.49 (41.62-43.37) |
|  | **Primary** | 53.98 (53.64-54.31) | 59.11 (57.37-60.84) | 38.58 (37.7-39.46) |
|  | **Secondary** | 53.54 (53.38-53.7) | 53.94 (53.17-54.71) | 43.11 (42.71-43.51) |
|  | **Higher** | 54.45 (54.15-54.76) | 52.47 (51.03-53.9) | 43.41 (42.69-44.14) |
| **Meat/fish** | **No education** | 58.08 (57.85-58.32) | 60.47 (59.06-61.87) | 41.54 (40.67-42.41) |
|  | **Primary** | 52.7 (52.37-53.04) | 56.72 (54.97-58.46) | 37.38 (36.5-38.26) |
|  | **Secondary** | 53.32 (53.16-53.48) | 54.04 (53.27-54.81) | 43.25 (42.86-43.65) |
|  | **Higher** | 56.89 (56.59-57.2) | 55.04 (53.62-56.47) | 46.33 (45.6-47.07) |

**Supplementary table 5:** Odds ratios (95% confidence intervals) for food group consumption by women in 2016 and 2021. Note: The reference categories are highest wealth quintile and higher education.

|  |  | **2016** | **2021** |
| --- | --- | --- | --- |
| **Dairy** | **Lowest** | 5.61 (5.5-5.72) | 5.95 (5.83-6.07) |
|  | **Low** | 3.38 (3.32-3.44) | 3.3 (3.24-3.37) |
|  | **Middle** | 2.33 (2.28-2.37) | 2.19 (2.14-2.23) |
|  | **High** | 1.66 (1.63-1.69) | 1.58 (1.54-1.61) |
|  | **No education** | 1.39 (1.36-1.42) | 1.15 (1.13-1.18) |
|  | **Primary** | 1.52 (1.49-1.56) | 1.35 (1.32-1.38) |
|  | **Secondary** | 1.38 (1.36-1.41) | 1.26 (1.23-1.28) |
| **Pulses/legumes/beans** | **Lowest** | 2.19 (2.13-2.25) | 2.1 (2.03-2.16) |
|  | **Low** | 1.88 (1.83-1.93) | 1.84 (1.79-1.9) |
|  | **Middle** | 1.64 (1.59-1.68) | 1.61 (1.56-1.66) |
|  | **High** | 1.4 (1.36-1.44) | 1.37 (1.33-1.41) |
|  | **No education** | 1.33 (1.29-1.37) | 0.99 (0.96-1.02) |
|  | **Primary** | 1.28 (1.24-1.33) | 1.09 (1.05-1.13) |
|  | **Secondary** | 1.23 (1.2-1.27) | 1.09 (1.06-1.12) |
| **Dark leafy green vegetables** | **Lowest** | 1.22 (1.19-1.25) | 1.17 (1.13-1.2) |
|  | **Low** | 1.07 (1.04-1.09) | 1.19 (1.15-1.22) |
|  | **Middle** | 1.02 (1-1.05) | 1.23 (1.2-1.27) |
|  | **High** | 1.02 (1-1.05) | 1.22 (1.18-1.25) |
|  | **No education** | 1.6 (1.55-1.64) | 1.19 (1.15-1.22) |
|  | **Primary** | 1.26 (1.22-1.3) | 1.08 (1.04-1.11) |
|  | **Secondary** | 1.07 (1.04-1.1) | 1.07 (1.04-1.1) |
| **Vitamin-A rich fruits** | **Lowest** | 7.63 (7.48-7.78) | 5.09 (5-5.18) |
|  | **Low** | 4.37 (4.29-4.44) | 3.36 (3.31-3.42) |
|  | **Middle** | 2.86 (2.82-2.91) | 2.43 (2.39-2.47) |
|  | **High** | 1.84 (1.81-1.87) | 1.71 (1.69-1.74) |
|  | **No education** | 2.26 (2.21-2.3) | 2.07 (2.04-2.11) |
|  | **Primary** | 1.9 (1.86-1.95) | 1.82 (1.78-1.86) |
|  | **Secondary** | 1.52 (1.5-1.55) | 1.43 (1.41-1.45) |
| **Eggs** | **Lowest** | 1.01 (0.99-1.03) | 0.82 (0.81-0.83) |
|  | **Low** | 0.8 (0.79-0.81) | 0.74 (0.72-0.75) |
|  | **Middle** | 0.71 (0.69-0.72) | 0.68 (0.67-0.69) |
|  | **High** | 0.7 (0.69-0.71) | 0.72 (0.71-0.73) |
|  | **No education** | 1.34 (1.31-1.37) | 1.26 (1.24-1.28) |
|  | **Primary** | 1.1 (1.08-1.12) | 1.14 (1.11-1.16) |
|  | **Secondary** | 1.01 (0.99-1.02) | 1.05 (1.03-1.06) |
| **Meat/fish** | **Lowest** | 0.99 (0.97-1) | 0.66 (0.65-0.67) |
|  | **Low** | 0.76 (0.74-0.77) | 0.62 (0.61-0.63) |
|  | **Middle** | 0.67 (0.66-0.68) | 0.6 (0.59-0.61) |
|  | **High** | 0.66 (0.65-0.67) | 0.66 (0.65-0.67) |
|  | **No education** | 1.26 (1.23-1.28) | 1.24 (1.22-1.26) |
|  | **Primary** | 1.04 (1.02-1.06) | 1.08 (1.06-1.1) |
|  | **Secondary** | 0.95 (0.93-0.96) | 0.99 (0.97-1) |

**Supplementary table 6:** Odds ratios (95% confidence intervals) for food group consumption by pregnant women in 2016 and 2021. Note: The reference categories are highest wealth quintile and higher education.

|  |  | **2016** | **2021** |
| --- | --- | --- | --- |
| **Dairy** | **Lowest** | 6.85 (6.14-7.63) | 8.02 (7.07-9.09) |
|  | **Low** | 4.24 (3.82-4.71) | 4.18 (3.69-4.73) |
|  | **Middle** | 2.85 (2.56-3.17) | 2.46 (2.16-2.8) |
|  | **High** | 1.82 (1.63-2.03) | 1.76 (1.55-2.02) |
|  | **No education** | 1.86 (1.66-2.09) | 1.38 (1.23-1.55) |
|  | **Primary** | 2.04 (1.81-2.3) | 1.53 (1.35-1.72) |
|  | **Secondary** | 1.68 (1.51-1.87) | 1.36 (1.23-1.5) |
| **Pulses/legumes/beans** | **Lowest** | 2.01 (1.75-2.31) | 1.63 (1.39-1.9) |
|  | **Low** | 1.74 (1.52-1.99) | 1.44 (1.23-1.68) |
|  | **Middle** | 1.52 (1.33-1.75) | 1.41 (1.21-1.64) |
|  | **High** | 1.31 (1.14-1.51) | 1.1 (0.94-1.29) |
|  | **No education** | 1.52 (1.31-1.77) | 1.17 (0.99-1.38) |
|  | **Primary** | 1.42 (1.21-1.67) | 1.34 (1.13-1.59) |
|  | **Secondary** | 1.4 (1.22-1.61) | 1.24 (1.08-1.41) |
| **Dark leafy green vegetables** | **Lowest** | 0.91 (0.81-1.02) | 1.14 (0.97-1.34) |
|  | **Low** | 0.88 (0.78-0.98) | 1.14 (0.97-1.33) |
|  | **Middle** | 0.86 (0.77-0.97) | 1.21 (1.04-1.41) |
|  | **High** | 0.92 (0.82-1.03) | 1.13 (0.97-1.33) |
|  | **No education** | 2.19 (1.92-2.5) | 1.22 (1.03-1.43) |
|  | **Primary** | 1.74 (1.51-2) | 1.13 (0.95-1.35) |
|  | **Secondary** | 1.23 (1.09-1.38) | 1.03 (0.91-1.18) |
| **Vitamin-A rich fruits** | **Lowest** | 10.03 (9.09-11.06) | 6.32 (5.7-7.02) |
|  | **Low** | 5.22 (4.77-5.71) | 3.72 (3.36-4.12) |
|  | **Middle** | 3.05 (2.79-3.33) | 2.6 (2.35-2.89) |
|  | **High** | 1.87 (1.71-2.05) | 1.71 (1.53-1.9) |
|  | **No education** | 2.51 (2.27-2.78) | 2.38 (2.15-2.63) |
|  | **Primary** | 2.28 (2.05-2.54) | 2.04 (1.83-2.27) |
|  | **Secondary** | 1.65 (1.51-1.8) | 1.49 (1.37-1.62) |
| **Eggs** | **Lowest** | 0.95 (0.87-1.04) | 0.8 (0.73-0.87) |
|  | **Low** | 0.76 (0.7-0.82) | 0.73 (0.67-0.79) |
|  | **Middle** | 0.74 (0.68-0.81) | 0.7 (0.65-0.77) |
|  | **High** | 0.77 (0.71-0.84) | 0.73 (0.67-0.79) |
|  | **No education** | 1.42 (1.3-1.56) | 1.35 (1.23-1.48) |
|  | **Primary** | 1.22 (1.1-1.35) | 1.26 (1.14-1.39) |
|  | **Secondary** | 1.02 (0.95-1.11) | 1.12 (1.05-1.21) |
| **Meat/fish** | **Lowest** | 0.93 (0.85-1.02) | 0.61 (0.55-0.66) |
|  | **Low** | 0.74 (0.68-0.81) | 0.61 (0.56-0.67) |
|  | **Middle** | 0.7 (0.64-0.76) | 0.64 (0.58-0.69) |
|  | **High** | 0.7 (0.65-0.76) | 0.64 (0.59-0.69) |
|  | **No education** | 1.26 (1.15-1.38) | 1.4 (1.28-1.54) |
|  | **Primary** | 1.08 (0.98-1.19) | 1.22 (1.11-1.35) |
|  | **Secondary** | 0.93 (0.86-1.01) | 1.1 (1.02-1.18) |

**Supplementary table 7:** Odds ratios (95% confidence intervals) for food group consumption by men in 2016 and 2021. Note: The reference categories are highest wealth quintile and higher education.

|  |  | **2016** | **2021** |
| --- | --- | --- | --- |
| **Dairy** | **Lowest** | 5.91 (5.61-6.23) | 7.1 (6.67-7.55) |
|  | **Low** | 3.49 (3.33-3.67) | 4.05 (3.81-4.3) |
|  | **Middle** | 2.33 (2.21-2.45) | 2.68 (2.52-2.85) |
|  | **High** | 1.71 (1.62-1.8) | 1.75 (1.64-1.87) |
|  | **No education** | 1.41 (1.34-1.5) | 1.23 (1.16-1.31) |
|  | **Primary** | 1.51 (1.43-1.6) | 1.34 (1.26-1.42) |
|  | **Secondary** | 1.27 (1.21-1.33) | 1.17 (1.12-1.23) |
| **Pulses/legumes/beans** | **Lowest** | 2.14 (1.99-2.29) | 2.28 (2.09-2.48) |
|  | **Low** | 1.67 (1.56-1.78) | 1.81 (1.66-1.97) |
|  | **Middle** | 1.62 (1.52-1.74) | 1.62 (1.49-1.77) |
|  | **High** | 1.49 (1.4-1.6) | 1.45 (1.33-1.58) |
|  | **No education** | 1.33 (1.23-1.43) | 1.39 (1.27-1.52) |
|  | **Primary** | 1.16 (1.08-1.26) | 1.23 (1.13-1.35) |
|  | **Secondary** | 1.17 (1.1-1.24) | 1.15 (1.08-1.24) |
| **Dark leafy green vegetables** | **Lowest** | 1.73 (1.62-1.84) | 1.28 (1.18-1.39) |
|  | **Low** | 1.18 (1.11-1.25) | 1.16 (1.08-1.26) |
|  | **Middle** | 1.12 (1.06-1.2) | 1.15 (1.06-1.24) |
|  | **High** | 1.1 (1.03-1.16) | 1.18 (1.1-1.28) |
|  | **No education** | 1.34 (1.25-1.45) | 1.47 (1.35-1.6) |
|  | **Primary** | 1.2 (1.11-1.29) | 1.13 (1.03-1.23) |
|  | **Secondary** | 1.13 (1.06-1.19) | 1.11 (1.04-1.18) |
| **Vitamin-A rich fruits** | **Lowest** | 5.71 (5.45-5.98) | 4.26 (4.07-4.47) |
|  | **Low** | 3.3 (3.17-3.44) | 2.85 (2.72-2.97) |
|  | **Middle** | 2.28 (2.2-2.38) | 2.11 (2.02-2.21) |
|  | **High** | 1.6 (1.54-1.67) | 1.6 (1.53-1.67) |
|  | **No education** | 1.7 (1.62-1.79) | 1.62 (1.54-1.71) |
|  | **Primary** | 1.65 (1.57-1.74) | 1.48 (1.41-1.56) |
|  | **Secondary** | 1.37 (1.32-1.42) | 1.29 (1.24-1.33) |
| **Eggs** | **Lowest** | 1.15 (1.1-1.2) | 0.98 (0.94-1.02) |
|  | **Low** | 0.9 (0.86-0.93) | 0.83 (0.8-0.87) |
|  | **Middle** | 0.82 (0.79-0.85) | 0.75 (0.72-0.78) |
|  | **High** | 0.77 (0.74-0.8) | 0.74 (0.71-0.77) |
|  | **No education** | 1.07 (1.02-1.13) | 1.08 (1.02-1.13) |
|  | **Primary** | 0.99 (0.95-1.04) | 0.96 (0.91-1.01) |
|  | **Secondary** | 1.07 (1.03-1.11) | 1.07 (1.04-1.11) |
| **Meat/fish** | **Lowest** | 1.09 (1.05-1.14) | 0.7 (0.67-0.73) |
|  | **Low** | 0.83 (0.8-0.87) | 0.63 (0.61-0.66) |
|  | **Middle** | 0.76 (0.73-0.79) | 0.62 (0.59-0.64) |
|  | **High** | 0.71 (0.69-0.74) | 0.63 (0.61-0.66) |
|  | **No education** | 0.96 (0.91-1) | 1.01 (0.96-1.06) |
|  | **Primary** | 0.88 (0.84-0.93) | 0.9 (0.86-0.95) |
|  | **Secondary** | 1 (0.97-1.04) | 1.04 (1.01-1.08) |

**Supplementary table 8:** Odds ratios (95% confidence intervals) for food group consumption by women

|  |  | **Dairy** | **Pulses/legumes/beans** | **Dark leafy green vegetables** | **Vitamin-A rich fruits** | **Eggs** | **Meat/fish** |
| --- | --- | --- | --- | --- | --- | --- | --- |
| **Year** | **2021** | 0.82 (0.79-0.84) | 0.81 (0.78-0.85) | 0.62 (0.6-0.64) | 0.94 (0.92-0.96) | 0.83 (0.81-0.85) | 0.94 (0.92-0.96) |
| **Wealth** | **Lowest** | 5.61 (5.5-5.72) | 2.19 (2.13-2.25) | 1.22 (1.19-1.25) | 7.63 (7.48-7.78) | 1.01 (0.99-1.03) | 0.99 (0.97-1) |
|  | **Low** | 3.38 (3.32-3.44) | 1.88 (1.83-1.93) | 1.07 (1.04-1.09) | 4.37 (4.29-4.44) | 0.8 (0.79-0.81) | 0.76 (0.74-0.77) |
|  | **Middle** | 2.33 (2.28-2.37) | 1.64 (1.59-1.68) | 1.02 (1-1.05) | 2.86 (2.82-2.91) | 0.71 (0.69-0.72) | 0.67 (0.66-0.68) |
|  | **High** | 1.66 (1.63-1.69) | 1.4 (1.36-1.44) | 1.02 (1-1.05) | 1.84 (1.81-1.87) | 0.7 (0.69-0.71) | 0.66 (0.65-0.67) |
| **Education** | **No education** | 1.39 (1.36-1.42) | 1.33 (1.29-1.37) | 1.6 (1.55-1.64) | 2.26 (2.21-2.3) | 1.34 (1.31-1.37) | 1.26 (1.23-1.28) |
|  | **Primary** | 1.52 (1.49-1.56) | 1.28 (1.24-1.33) | 1.26 (1.22-1.3) | 1.9 (1.86-1.95) | 1.1 (1.08-1.12) | 1.04 (1.02-1.06) |
|  | **Secondary** | 1.38 (1.36-1.41) | 1.23 (1.2-1.27) | 1.07 (1.04-1.1) | 1.52 (1.5-1.55) | 1.01 (0.99-1.02) | 0.95 (0.93-0.96) |
| **Year*wealth** | **Lowest** | 1.06 (1.03-1.09) | 0.96 (0.92-1) | 0.96 (0.92-1) | 0.67 (0.65-0.68) | 0.81 (0.79-0.83) | 0.67 (0.65-0.69) |
|  | **Low** | 0.98 (0.95-1.01) | 0.98 (0.94-1.02) | 1.11 (1.07-1.15) | 0.77 (0.75-0.79) | 0.92 (0.9-0.94) | 0.82 (0.8-0.84) |
|  | **Middle** | 0.94 (0.91-0.97) | 0.98 (0.94-1.02) | 1.2 (1.16-1.25) | 0.85 (0.83-0.87) | 0.96 (0.94-0.98) | 0.9 (0.88-0.92) |
|  | **High** | 0.95 (0.92-0.98) | 0.98 (0.94-1.02) | 1.19 (1.15-1.24) | 0.93 (0.91-0.95) | 1.02 (1-1.04) | 0.99 (0.97-1.02) |
| **Year*education** | **No education** | 0.83 (0.8-0.85) | 0.75 (0.71-0.78) | 0.74 (0.71-0.77) | 0.92 (0.89-0.94) | 0.94 (0.91-0.96) | 0.99 (0.97-1.02) |
|  | **Primary** | 0.89 (0.86-0.92) | 0.85 (0.81-0.89) | 0.86 (0.82-0.89) | 0.96 (0.93-0.98) | 1.03 (1-1.06) | 1.04 (1.01-1.07) |
|  | **Secondary** | 0.91 (0.88-0.93) | 0.88 (0.85-0.92) | 1 (0.96-1.03) | 0.94 (0.92-0.96) | 1.04 (1.02-1.06) | 1.04 (1.02-1.07) |
| **Constant** | | 0.17 (0.17-0.17) | 0.07 (0.07-0.07) | 0.13 (0.13-0.13) | 0.29 (0.29-0.3) | 1.9 (1.87-1.93) | 1.9 (1.87-1.93) |

Note: The reference categories are highest wealth quintile and higher education. The global p-values were less than 0.01 for both the year/wealth and year/education interactions for every food group. The only exception to this was a p-value of 0.237 of the year/wealth interaction for pulses/legumes/beans.

**Supplementary table 9:** Odds ratios (95% confidence intervals) for food group consumption by pregnant women

|  |  | **Dairy** | **Pulses/legumes/beans** | **Dark leafy green vegetables** | **Vitamin-A rich fruits** | **Eggs** | **Meat/fish** |
| --- | --- | --- | --- | --- | --- | --- | --- |
| **Year** | **2021** | 0.91 (0.77-1.07) | 1.01 (0.83-1.23) | 0.57 (0.48-0.68) | 0.86 (0.75-0.98) | 0.72 (0.65-0.8) | 0.81 (0.73-0.89) |
| **Wealth** | **Lowest** | 6.85 (6.14-7.63) | 2.01 (1.75-2.31) | 0.91 (0.81-1.02) | 10.03 (9.09-11.06) | 0.95 (0.87-1.04) | 0.93 (0.85-1.02) |
|  | **Low** | 4.24 (3.82-4.71) | 1.74 (1.52-1.99) | 0.88 (0.78-0.98) | 5.22 (4.77-5.71) | 0.76 (0.7-0.82) | 0.74 (0.68-0.81) |
|  | **Middle** | 2.85 (2.56-3.17) | 1.52 (1.33-1.75) | 0.86 (0.77-0.97) | 3.05 (2.79-3.33) | 0.74 (0.68-0.81) | 0.7 (0.64-0.76) |
|  | **High** | 1.82 (1.63-2.03) | 1.31 (1.14-1.51) | 0.92 (0.82-1.03) | 1.87 (1.71-2.05) | 0.77 (0.71-0.84) | 0.7 (0.65-0.76) |
| **Education** | **No education** | 1.86 (1.66-2.09) | 1.52 (1.31-1.77) | 2.19 (1.92-2.5) | 2.51 (2.27-2.78) | 1.42 (1.3-1.56) | 1.26 (1.15-1.38) |
|  | **Primary** | 2.04 (1.81-2.3) | 1.42 (1.21-1.67) | 1.74 (1.51-2) | 2.28 (2.05-2.54) | 1.22 (1.1-1.35) | 1.08 (0.98-1.19) |
|  | **Secondary** | 1.68 (1.51-1.87) | 1.4 (1.22-1.61) | 1.23 (1.09-1.38) | 1.65 (1.51-1.8) | 1.02 (0.95-1.11) | 0.93 (0.86-1.01) |
| **Year*wealth** | **Lowest** | 1.17 (0.99-1.38) | 0.81 (0.66-0.99) | 1.26 (1.03-1.53) | 0.63 (0.55-0.73) | 0.84 (0.74-0.95) | 0.65 (0.58-0.74) |
|  | **Low** | 0.98 (0.84-1.16) | 0.83 (0.67-1.01) | 1.3 (1.07-1.58) | 0.71 (0.62-0.82) | 0.96 (0.85-1.08) | 0.82 (0.73-0.93) |
|  | **Middle** | 0.86 (0.73-1.02) | 0.92 (0.75-1.13) | 1.41 (1.16-1.7) | 0.85 (0.75-0.98) | 0.95 (0.84-1.07) | 0.91 (0.81-1.03) |
|  | **High** | 0.97 (0.82-1.15) | 0.84 (0.68-1.04) | 1.23 (1.01-1.49) | 0.91 (0.79-1.05) | 0.94 (0.84-1.06) | 0.91 (0.8-1.02) |
| **Year*education** | **No education** | 0.74 (0.63-0.87) | 0.77 (0.62-0.96) | 0.56 (0.45-0.68) | 0.95 (0.82-1.09) | 0.95 (0.83-1.08) | 1.12 (0.98-1.27) |
|  | **Primary** | 0.75 (0.63-0.89) | 0.95 (0.75-1.2) | 0.65 (0.52-0.81) | 0.89 (0.77-1.04) | 1.04 (0.9-1.19) | 1.13 (0.98-1.3) |
|  | **Secondary** | 0.81 (0.7-0.93) | 0.88 (0.73-1.07) | 0.84 (0.71-1) | 0.9 (0.8-1.02) | 1.1 (0.99-1.22) | 1.18 (1.06-1.31) |
| **Constant** | | 0.09 (0.08-0.1) | 0.07 (0.06-0.08) | 0.13 (0.12-0.15) | 0.17 (0.16-0.19) | 1.98 (1.84-2.13) | 2.04 (1.9-2.2) |

Note: The reference categories are highest wealth quintile and higher education. The global p-values were less than 0.01 for both the year/wealth and year/education interactions for pregnant women not consuming dairy. The global p-value for the year/wealth interaction was 0.18 and 0.05 for the year/education interaction for pregnant women not consuming pulses/legumes/beans. The global p-value for the year/wealth interaction was 0.01 and less than 0.01 for the year/education interaction for pregnant women not consuming dark leafy green vegetables. The global p-value for the year/wealth interaction was less than 0.01 and 0.28 for the year/education for pregnant women not consuming vitamin-A rich fruits. The global p-value for the year/wealth interaction was 0.04 and 0.01 for the year/education interaction for pregnant women not consuming eggs. The global p-value for the year/wealth interaction was less than 0.01 and 0.02 for the year/education interaction for pregnant women not consuming meat/fish.

**Supplementary table 10:** Odds ratios (95% confidence intervals) for food group consumption by men

|  |  | **Dairy** | **Pulses/legumes/beans** | **Dark leafy green vegetables** | **Vitamin-A rich fruits** | **Eggs** | **Meat/fish** |
| --- | --- | --- | --- | --- | --- | --- | --- |
| **Year** | **2021** | 0.73 (0.67-0.79) | 0.7 (0.63-0.78) | 0.7 (0.64-0.77) | 0.92 (0.87-0.97) | 0.87 (0.83-0.91) | 1.06 (1-1.11) |
| **Wealth** | **Lowest** | 5.91 (5.61-6.23) | 2.14 (1.99-2.29) | 1.73 (1.62-1.84) | 5.71 (5.45-5.98) | 1.15 (1.1-1.2) | 1.09 (1.05-1.14) |
|  | **Low** | 3.49 (3.33-3.67) | 1.67 (1.56-1.78) | 1.18 (1.11-1.25) | 3.3 (3.17-3.44) | 0.9 (0.86-0.93) | 0.83 (0.8-0.87) |
|  | **Middle** | 2.33 (2.21-2.45) | 1.62 (1.52-1.74) | 1.12 (1.06-1.2) | 2.28 (2.2-2.38) | 0.82 (0.79-0.85) | 0.76 (0.73-0.79) |
|  | **High** | 1.71 (1.62-1.8) | 1.49 (1.4-1.6) | 1.1 (1.03-1.16) | 1.6 (1.54-1.67) | 0.77 (0.74-0.8) | 0.71 (0.69-0.74) |
| **Education** | **No education** | 1.41 (1.34-1.5) | 1.33 (1.23-1.43) | 1.34 (1.25-1.45) | 1.7 (1.62-1.79) | 1.07 (1.02-1.13) | 0.96 (0.91-1) |
|  | **Primary** | 1.51 (1.43-1.6) | 1.16 (1.08-1.26) | 1.2 (1.11-1.29) | 1.65 (1.57-1.74) | 0.99 (0.95-1.04) | 0.88 (0.84-0.93) |
|  | **Secondary** | 1.27 (1.21-1.33) | 1.17 (1.1-1.24) | 1.13 (1.06-1.19) | 1.37 (1.32-1.42) | 1.07 (1.03-1.11) | 1 (0.97-1.04) |
| **Year*wealth** | **Lowest** | 1.2 (1.11-1.3) | 1.07 (0.95-1.19) | 0.74 (0.67-0.82) | 0.75 (0.7-0.8) | 0.85 (0.8-0.9) | 0.64 (0.6-0.68) |
|  | **Low** | 1.16 (1.07-1.25) | 1.09 (0.98-1.21) | 0.99 (0.9-1.09) | 0.86 (0.81-0.92) | 0.92 (0.87-0.98) | 0.76 (0.72-0.8) |
|  | **Middle** | 1.15 (1.06-1.25) | 1 (0.9-1.11) | 1.02 (0.93-1.13) | 0.92 (0.87-0.98) | 0.92 (0.87-0.97) | 0.82 (0.77-0.86) |
|  | **High** | 1.03 (0.95-1.12) | 0.97 (0.87-1.08) | 1.08 (0.98-1.19) | 1 (0.94-1.06) | 0.97 (0.92-1.02) | 0.88 (0.84-0.93) |
| **Year*education** | **No education** | 0.87 (0.8-0.95) | 1.05 (0.93-1.18) | 1.09 (0.97-1.22) | 0.95 (0.89-1.03) | 1 (0.94-1.07) | 1.05 (0.98-1.13) |
|  | **Primary** | 0.88 (0.81-0.96) | 1.06 (0.94-1.2) | 0.94 (0.84-1.06) | 0.9 (0.83-0.96) | 0.97 (0.9-1.04) | 1.02 (0.96-1.1) |
|  | **Secondary** | 0.92 (0.86-0.99) | 0.99 (0.9-1.09) | 0.98 (0.9-1.07) | 0.94 (0.89-0.99) | 1 (0.95-1.05) | 1.04 (0.99-1.1) |
| **Constant** | | 0.12 (0.12-0.13) | 0.07 (0.06-0.07) | 0.1 (0.1-0.11) | 0.34 (0.33-0.36) | 1.26 (1.22-1.31) | 1.39 (1.35-1.44) |

Note: The reference categories are highest wealth quintile and higher education. The global p-values were less than 0.01 for both the year/wealth and year/education interactions for men not consuming dairy. The global p-value for the year/wealth interaction was 0.08 and 0.35 for the year/education interaction for men not consuming pulses/legumes/beans. The global p-value for the year/wealth interaction was less than 0.01 and 0.04 for the year/education interaction for men not consuming dark leafy green vegetables. The global p-value for the year/wealth interaction was less than 0.01 and 0.02 for the year/education interaction for men not consuming vitamin-A rich fruits. The global p-value for the year/wealth interaction was less than 0.01 and 0.65 for the year/education interaction for men not consuming eggs. The global p-value for the year/wealth interaction was less than 0.01 and 0.33 for the year/education interaction for men not consuming meat/fish.

**Supplementary table 11:** Percent (95% confidence interval) of women and men (couples) in the same household not consuming a food group by household wealth quintile

|  |  | **2016** | | | | **2021** | | | |
| --- | --- | --- | --- | --- | --- | --- | --- | --- | --- |
|  |  | **Women** | **Men** | **Wives** | **Husbands** | **Women** | **Men** | **Wives** | **Husbands** |
| **Dairy** | **Lowest** | 54.36 (54.09-54.63) | 47.71 (46.99-48.44) | 55.93 (55.02-56.84) | 49.8 (48.89-50.72) | 47.42 (47.16-47.67) | 38.07 (37.4-38.75) | 48.54 (47.64-49.44) | 39.37 (38.49-40.25) |
|  | **Highest** | 14.53 (14.34-14.72) | 11.88 (11.46-12.3) | 14.52 (13.91-15.14) | 11.81 (11.25-12.38) | 13.79 (13.59-13.98) | 8.46 (8.05-8.87) | 14.17 (13.48-14.87) | 8.17 (7.63-8.72) |
| **Pulses/legumes/beans** | **Lowest** | 14.29 (14.11-14.48) | 13.34 (12.85-13.83) | 14.08 (13.44-14.71) | 13.32 (12.69-13.94) | 8.71 (8.56-8.85) | 9.19 (8.79-9.6) | 8.82 (8.31-9.33) | 8.79 (8.28-9.3) |
|  | **Highest** | 6.41 (6.28-6.55) | 7.02 (6.69-7.36) | 6.4 (5.97-6.83) | 6.56 (6.12-6.99) | 5.31 (5.19-5.44) | 4.95 (4.63-5.27) | 5.07 (4.64-5.51) | 4.31 (3.9-4.71) |
| **Dark leafy green vegetables** | **Lowest** | 17.06 (16.86-17.27) | 16.14 (15.61-16.67) | 17.22 (16.53-17.91) | 16.02 (15.34-16.69) | 8.95 (8.8-9.09) | 8.48 (8.1-8.87) | 8.58 (8.08-9.08) | 7.71 (7.23-8.19) |
|  | **Highest** | 12.37 (12.19-12.55) | 10.1 (9.71-10.49) | 11.53 (10.97-12.09) | 9.64 (9.12-10.16) | 8.08 (7.93-8.23) | 6.79 (6.41-7.16) | 7.71 (7.18-8.24) | 6.74 (6.24-7.24) |
| **Vitamin-A rich fruits** | **Lowest** | 81.02 (80.8-81.23) | 74.64 (74.01-75.27) | 81.93 (81.23-82.64) | 75.91 (75.12-76.69) | 73.18 (72.96-73.4) | 63.27 (62.6-63.95) | 73.04 (72.25-73.84) | 65.14 (64.28-66) |
|  | **Highest** | 26.03 (25.8-26.27) | 28.88 (28.3-29.47) | 25.88 (25.11-26.64) | 29.02 (28.22-29.82) | 26.3 (26.06-26.55) | 25.79 (25.14-26.43) | 27.06 (26.17-27.95) | 25.32 (24.46-26.19) |
| **Eggs** | **Lowest** | 67.51 (67.25-67.76) | 57.84 (57.12-58.55) | 66.69 (65.82-67.55) | 57.57 (56.66-58.47) | 58.59 (58.34-58.84) | 44.91 (44.22-45.6) | 56.66 (55.77-57.55) | 44.83 (43.93-45.72) |
|  | **Highest** | 60.28 (60.02-60.55) | 53.02 (52.37-53.67) | 60.3 (59.44-61.15) | 53.11 (52.24-53.99) | 57.77 (57.5-58.05) | 45.7 (44.96-46.44) | 58.06 (57.08-59.05) | 47.9 (46.91-48.9) |
| **Meat/fish** | **Lowest** | 65.82 (65.57-66.08) | 57.33 (56.62-58.05) | 64.88 (64-65.75) | 56.82 (55.91-57.73) | 56.05 (55.8-56.3) | 41.22 (40.54-41.91) | 52.67 (51.77-53.56) | 40.12 (39.24-41) |
|  | **Highest** | 59.58 (59.32-59.85) | 54.65 (54-55.29) | 58.89 (58.03-59.75) | 54.88 (54-55.75) | 60.11 (59.83-60.38) | 49.13 (48.39-49.87) | 60.29 (59.32-61.27) | 51.23 (50.23-52.22) |

**Supplementary table 12:** Percent (95% confidence interval) of women and men (couples) in the same household not consuming a food group by education

|  |  | **2016** | | | | **2021** | | | |  |
| --- | --- | --- | --- | --- | --- | --- | --- | --- | --- | --- |
|  |  | **Women** | **Men** | **Wives** | **Husbands** | **Women** | **Men** | **Wives** | **Husbands** | |
| **Dairy** | **No education** | 41.3 (41.08-41.51) | 36.42 (35.65-37.19) | 40.88 (40.22-41.54) | 34 (33.36-34.64) | 33.96 (33.73-34.18) | 27.89 (27.1-28.69) | 34.38 (33.42-35.35) | 28.48 (27.56-29.4) | |
|  | **Higher education** | 16.31 (16.05-16.57) | 14.14 (13.62-14.65) | 14.44 (13.53-15.36) | 12.73 (11.86-13.6) | 16.52 (16.3-16.75) | 11.92 (11.44-12.39) | 17.34 (16.52-18.15) | 10.81 (10.14-11.48) | |
| **Pulses/legumes/beans** | **No education** | 12.47 (12.33-12.62) | 12.25 (11.73-12.78) | 12.2 (11.76-12.64) | 10.9 (10.48-11.32) | 7.86 (7.73-7.99) | 8.55 (8.06-9.05) | 7.97 (7.42-8.52) | 8.05 (7.5-8.61) | |
|  | **Higher education** | 6.92 (6.75-7.1) | 7.31 (6.93-7.7) | 5.95 (5.34-6.57) | 6.66 (6.01-7.32) | 5.72 (5.57-5.86) | 5.14 (4.81-5.46) | 5.68 (5.18-6.18) | 4.1 (3.67-4.53) | |
| **Dark leafy green vegetables** | **No education** | 18.32 (18.15-18.49) | 14.7 (14.14-15.27) | 18.9 (18.38-19.43) | 14.51 (14.04-14.99) | 9.94 (9.8-10.08) | 9.67 (9.14-10.19) | 10.15 (9.54-10.77) | 9.33 (8.74-9.93) | |
|  | **Higher education** | 11.87 (11.64-12.09) | 9.51 (9.08-9.94) | 10.91 (10.1-11.73) | 8.98 (8.24-9.73) | 8.41 (8.24-8.59) | 6.93 (6.56-7.31) | 7.48 (6.91-8.05) | 6.39 (5.86-6.92) | |
| **Vitamin-A rich fruits** | **No education** | 71.82 (71.62-72.02) | 64.82 (64.06-65.59) | 71.89 (71.28-72.49) | 64.52 (63.87-65.16) | 65.76 (65.54-65.99) | 57.33 (56.46-58.21) | 64.82 (63.85-65.79) | 56.93 (55.92-57.93) | |
|  | **Higher education** | 28.08 (27.77-28.39) | 32.34 (31.65-33.03) | 21.87 (20.79-22.95) | 26.25 (25.1-27.39) | 29.51 (29.23-29.79) | 29.74 (29.07-30.41) | 30.87 (29.88-31.87) | 27.77 (26.81-28.74) | |
| **Eggs** | **No education** | 64.32 (64.11-64.53) | 51.97 (51.17-52.77) | 65.53 (64.88-66.17) | 56.82 (56.16-57.49) | 58.81 (58.58-59.05) | 42.49 (41.62-43.37) | 52.14 (51.12-53.15) | 41.32 (40.32-42.32) | |
|  | **Higher education** | 57.31 (56.97-57.65) | 49.41 (48.66-50.15) | 55.31 (54.01-56.61) | 49.27 (47.96-50.57) | 54.45 (54.15-54.76) | 43.41 (42.69-44.14) | 57.66 (56.59-58.72) | 46.7 (45.63-47.78) | |
| **Meat/fish** | **No education** | 62.59 (62.38-62.81) | 50.56 (49.76-51.36) | 63.86 (63.22-64.51) | 56.62 (55.96-57.29) | 58.08 (57.85-58.32) | 41.54 (40.67-42.41) | 49.18 (48.16-50.19) | 40.81 (39.81-41.81) | |
|  | **Higher education** | 57.47 (57.13-57.81) | 52.07 (51.33-52.81) | 54.38 (53.08-55.68) | 51.07 (49.76-52.37) | 56.89 (56.59-57.2) | 46.33 (45.6-47.07) | 58.8 (57.74-59.86) | 49.04 (47.96-50.12) | |
